# Supplementary material for: BTLA-Expressing Memory B Cells Are Associated with Belimumab-Induced Improvement in Systemic Lupus Erythematosus
Source: Int J Mol Sci. 2025 Nov 23;26(23):11323. doi: 10.3390/ijms262311323 (PMC12692296; doi:10.3390/ijms262311323)
Supplement: Supplementary file 1 [file ijms-26-11323-s001.zip › ijms-3923859-supplementary.pdf]

Supplementary Material

BTLA-Expressing Memory B Cells Are Associated with Belimumab-Induced Improvement in Systemic Lupus Erythematosus

Takuya Nishi [1,2], Kunihiro Hayakawa [1], Keigo Ikeda [1,2,*], Maki Fujishiro [1], Yuko Kataoka [1], Ken Yamaji [3], Kenji Takamori [1], Naoto Tamura [3], Iwao Sekigawa [1,2,†] and Shinji Morimoto [1,2]

[1] Institute for Environment and Gender-Specific Medicine, Juntendo University Graduate School of Medicine, Chiba 279-0021, Japan; ta-nishi@juntendo.ac.jp (TN); khayaka@juntendo.ac.jp (KH); keigo@juntendo.ac.jp (KI); mfujishi@juntendo.ac.jp (MF); y-kataoka@juntendo.ac.jp (YK); ktakamor@juntendo.ac.jp (KT); morimoto@juntendo.ac.jp (SM)

[2] Department of Internal Medicine and Rheumatology, Juntendo University Urayasu Hospital, Chiba 279-0021, Japan

[3] Department of Internal Medicine and Rheumatology, School of Medicine, Juntendo University, Tokyo 113-8421, Japan; k.yamaji@juntendo.ac.jp (KY); tnaoto@juntendo.ac.jp (NT)

* Correspondence: Institutional address: 2-1-1 Tomioka Urayasu-shi, Chiba, Japan 279-0021; keigo@juntendo.ac.jp; Tel.: +81-47-353-3111; Fax: +81-47-381-5054

† Deceased: Principal Investigator Dr. Iwao Sekigawa passed away in August 2022.

Supplementary Figures

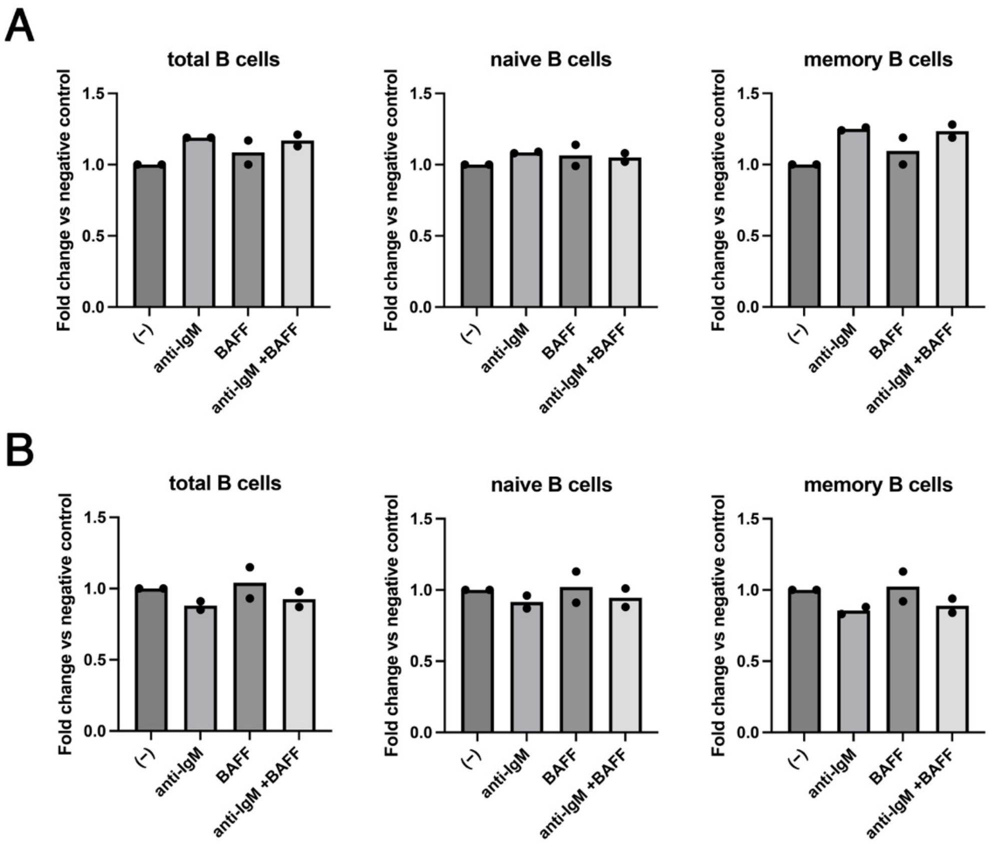

Figure S1. Effects of B cell receptor (BCR) signaling and B cell-activating factor (BAFF) stimulation on herpes virus entry mediator (HVEM) and B- and T-lymphocyte attenuator (BTLA) expression levels. (**A** and **B**) Isolated B cells from healthy controls (n = 2) were stimulated as indicated. Expression levels of (**A**) HVEM and (**B**) BTLA on total ($CD19^+$), naive ($CD19^+CD27^-$), and memory ($CD19^+CD27^+$) B cells were analyzed by flow cytometry. Each dot represents the fold change in mean fluorescence intensity (MFI) relative to the negative control from individual experiments, and bars indicate the mean.

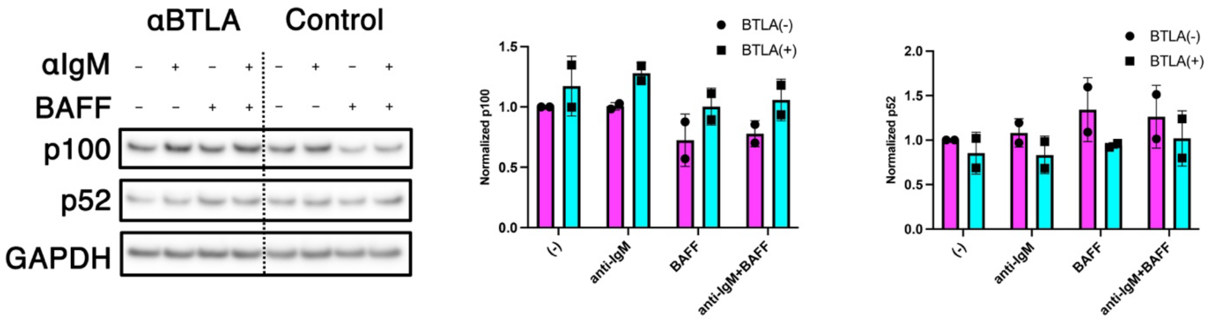

Figure S2. BTLA engagement tends to inhibit non-canonical nuclear factor (NF)-κB activation by increasing p100 levels and reducing p52 processing. Isolated B cells from healthy controls were incubated with the anti-BTLA mAb (αBTLA) or isotype controls and stimulated with or without anti-human IgM (αIgM) and human BAFF. Levels of signaling proteins were determined by western blotting. Glyceraldehyde-3-phosphate dehydrogenase (GAPDH) was used as a loading control. Left panel shows the representative protein bands of NF-κB2 (p100/p52) and GAPDH in western blotting. Middle and right panel shows the results of densitometric analysis of the bands in the left panel. NF-κB2 (p100/p52) band intensities were normalized to GAPDH. All assays were performed in duplicate. Each dot or square represents an individual experiment, and bars represent the mean ± standard deviation (SD).

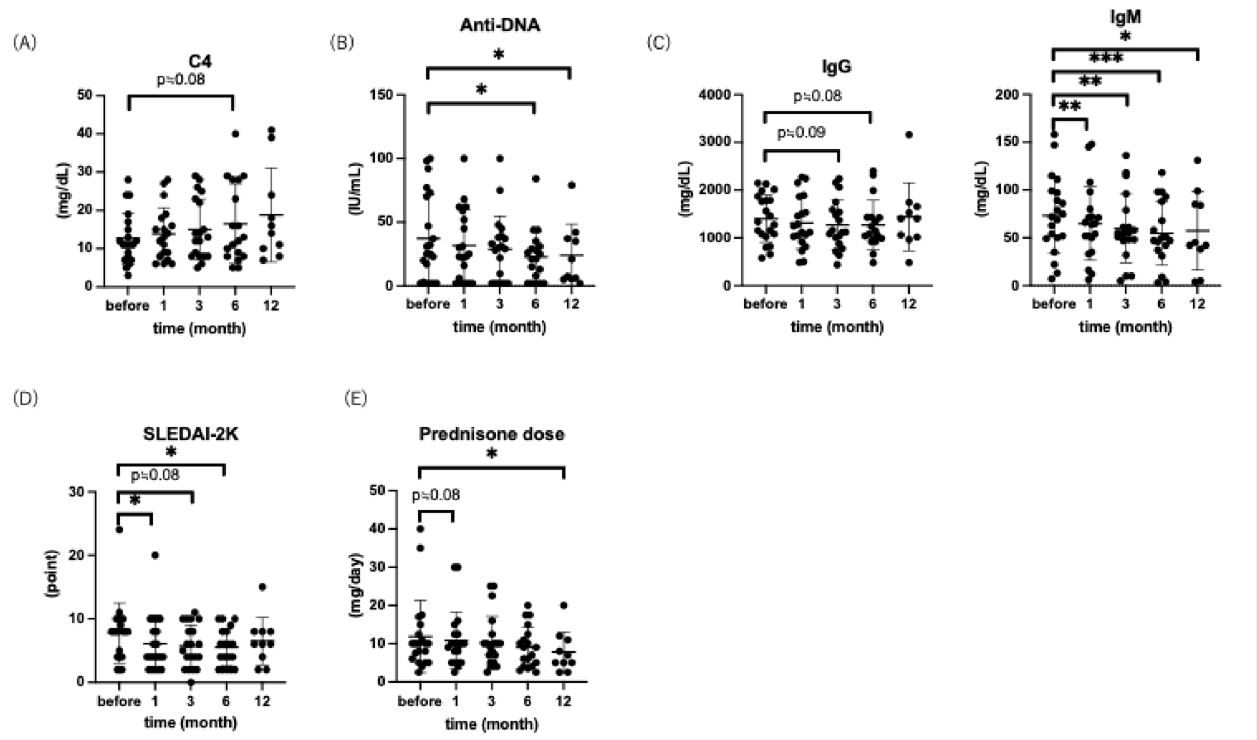

Figure S3. Serologic data, systemic lupus erythematosus (SLE) disease activity index 2000 (SLEDAI-2K) scores, and prednisone doses in patients SLE before and 1, 3, 6, and 12 months after belimumab treatment. (**A**) Serum complement C4 levels. (**B**) Anti-DNA antibodies titers. (**C**) Serum IgG and IgM levels. (**D**) SLEDAI-2K scores. (**E**) Prednisone doses. Each dot represents an individual patient sample, and bars represent the mean ± SD. Statistical analyses were performed using a mixed-effects model with Dunnett's post hoc test to compare the data before and 1, 3, 6, and 12 months after treatment. Statistically significant changes are marked with asterisks (*$p < 0.05$, **$p < 0.01$, and ***$p < 0.001$). Numbers on the graph indicate the p values.
